# Atmospheric–ocean coupling drives prevailing and synchronic dispersal patterns of marine species with long pelagic durations

**DOI:** 10.1038/s41598-023-29543-7

**Published:** 2023-02-09

**Authors:** Eduardo Ramirez-Romero, Angel Amores, David Diaz, Anabel Muñoz, Ignacio A. Catalan, Juan Carlos Molinero, Andres Ospina-Alvarez

**Affiliations:** 1 Instituto de Ciencias Marinas de Andalucía, ICMAN, CSIC, República Saharaui, 4, Puerto Real, 11519 Cádiz, Spain; 2 Department of Physics, University of the Balearic Islands, Crta. Valldemossa, km 7.5, 07122 Palma, Illes Balears Spain; 3 Mediterranean Institute for Advanced Studies, IMEDEA, CSIC-UIB, Miquel Marquès, 21, 07190 Esporles, Illes Balears Spain; 4 Instituto Español de Oceanografía, IEO, CSIC, Centre Oceanogràfic de les Balears, Moll de Ponent sn, 07015 Palma, Illes Balears Spain; 5 Marine Biodiversity Exploitation and Conservation, MARBEC, IRD, CNRS, Ifremer, Université de Montpellier, 610101 Sète, France

**Keywords:** Marine biology, Physical oceanography, Animal migration, Ecological modelling, Ecosystem services, Population dynamics

## Abstract

Dispersal shapes population connectivity and plays a critical role in marine metacommunities. Prominent species for coastal socioecological systems, such as jellyfish and spiny lobsters, feature long pelagic dispersal phases (LPDPs), which have long been overlooked. Here, we use a cross-scale approach combining field surveys of these species with a high-resolution hydrodynamic model to decipher the underlying mechanisms of LPDP patterns in northwestern Mediterranean shores. We identified basin-scale prevailing dispersal routes and synchronic year-to-year patterns tightly linked to prominent circulation features typical of marginal seas and semienclosed basins, with an outstanding role of a retentive source area replenishing shores and potentially acting as a pelagic nursery area. We show how the atmospheric forcing of the ocean, a marked hydrological driver of the Mediterranean Sea, modulates dispersal routes and sources of LPDP at interannual scales. These findings represent a crucial advance in our understanding of the functioning of metapopulations of species with LPDP in marginal seas and may contribute to the effective management of coastal ecosystem services in the face of climate change.

## Introduction

Marine populations inhabit a fragmented seascape where dispersal, i.e., the movement of organisms or their offspring away from their source to a settlement or nursery site, shapes gene flow and structures the connectivity and persistence of populations, thereby sculpting metacommunities^1^, dynamics and seashore biogeography^2^. Given the pivotal role of dispersal, a mechanistic understanding is essential for both theoretical (i.e., evolution) and management (i.e., conservation) reasons, as rapid anthropogenic impact is altering marine seascapes^3^, affecting species distribution and consequently nature’s contributions to people and human wellbeing^4^.

Nearshore ecosystems host a wide diversity of invertebrates featuring pelagic larval phases prone to dispersal by ocean currents. This process connects spawning locations with suitable nursery areas or settlement habitats. Dispersal takes place at multiple scales and is driven by a complex array of processes and interactions, namely, the lifespan of oceanographic features, temperature^5^, species life-history traits, including planktonic larval duration (PLD), and behavior (e.g., vertical migration)^6,7^. Efforts to unveil population connectivity have thus far mainly focused on short-term scales, $\approx$ 2–4 weeks^6^, using proxies combining PLD and genetic metrics^8^, and high-resolution biophysical coupled models^9^. Overall, at these scales, dispersion has been described as a highly stochastic and uncertain process^9^, while at long-term scales, the process remains less understood. Indeed, the dispersal and connectivity of species with LPDP (> 4 months) have long been overlooked, partly due to traditional research mainly focused on the late phase of dispersal, i.e., arrival at nursery and stranding areas, settlement and temporal correlations^10–12^, and partly due to the difficulty of making synoptic observations over broad spatial and temporal scales. Long-lasting plankton forms are shaped by interlinked biological, i.e., growth and mortality, and physical processes ranging from transient features, namely, fronts and mesoscale eddies, to basin-scale persistent physical phenomena, i.e., geostrophic currents^13^. Hence, dispersal and connectivity over broad scales may be nurtured by heterogeneous oceanic processes presenting different predictability levels^9,14–16^. In this regard, genetic studies can integrate to a certain extent the size and dynamics of metapopulations, including the effect of adults migrations (e.g. for fish)^17,18^ but may fail to capture underlying mechanisms linking hydrodynamic processes and species life cycles and derived interannual changes in population size. To fill this gap, we propose a complementary cross-scale study combining field surveys and state-of-the-art hydrodynamic modeling to reconstruct potential dispersal routes and entire dispersal kernels and to gain a mechanistic understanding of processes and scales shaping metapopulations.

In densely populated basins such as the Mediterranean Sea, some iconic species providing worthy natural contributions to people are featured by the LPDP. An example is the European spiny lobster (*Palinurus elephas*), a major target of artisanal fisheries since ancient times^19^, which faces overexploitation and is included in the red list of the International Union for Conservation of Nature’s Red List of Threatened Species^20^. Another prominent species also characterized by LPDP is the mauve stinger jellyfish (*Pelagia noctiluca*), which plays a key role in the pelagic food web and severely affects economic activities, including fisheries, aquaculture and tourism^21,22^. These species challenge conservation and management actions in the Mediterranean Sea, and therefore, resolving their dispersal patterns and connectivity has widespread implications for coastal communities and pivotal sectors. Tourism, particularly based on “beach and sun”, is an outstanding example in the Balearic Islands, contributing approximately 45% to the gross domestic product and 32% of jobs in the prepandemic scenario^23^.

We hypothesize that (i) due to the long pelagic lifespan of these species, large-scale persistent circulation features, typical of semienclosed basins, may play a prominent role in dispersal and connectivity in combination with stochastic processes. If this is true, (ii) dispersal patterns might be shared among different species with LPDP yielding synchronous interannual patterns. In addition, (iii) this physical-biological coupling may be intricately linked with the atmospheric forcing in the North Atlantic, acknowledged as a major driver of thermohaline circulation in the western Mediterranean^24,25^. The confirmation of these hypotheses might open opportunities for basin-scale conservation and management policies in this basin and might encourage analogous analyses in other marginal seas and semienclosed basins. To test these hypotheses, we used two model organisms with similar long pelagic lifespans. We combined the most comprehensive dataset of interannual records of both species in the Western Mediterranean shores and the state-of-the-art Lagrangian drifter scheme coupled with a high-resolution hydrodynamic model. We simulated back-in-time trajectories of particles to identify dispersal route pathways and source areas over a decade.

### Biological models

The European spiny lobster *P. elephas* is a valued, charismatic species that inhabits shallow rocky shores typical in Mediterranean ecosystems. By virtue of its long pelagic phase or phyllosomal larvae, ca. 8 months, compared to other meroplanktonic invertebrates, this species offers an excellent framework to assess mechanisms shaping long-term dispersal. The settlement of early benthic juveniles occurs in shallow rocky areas around late spring-early summer in this region, from May to August^26,27^.

The mauve stinger jellyfish is the most abundant holoplanktonic scyphozoan in the Mediterranean Sea, the main stinging species and one of the most worrying jellyfish due to its impact on human leisure and economic activities^21^. This species lives ca. 9 months^28^, being mainly associated with offshore nutrient-rich waters^29^, i.e., marine fronts and stranding events are closely linked with sea current variations^30,31^. Outbreaks of this species are associated with warm and dry late spring-early summers in the western Mediterranean^32^, although recent investigations have shown an enhanced bloom frequency and a permanent presence of *P. noctiluca* in the northernmost basin areas^33,34^.

### Physical oceanography

**Figure 1:**
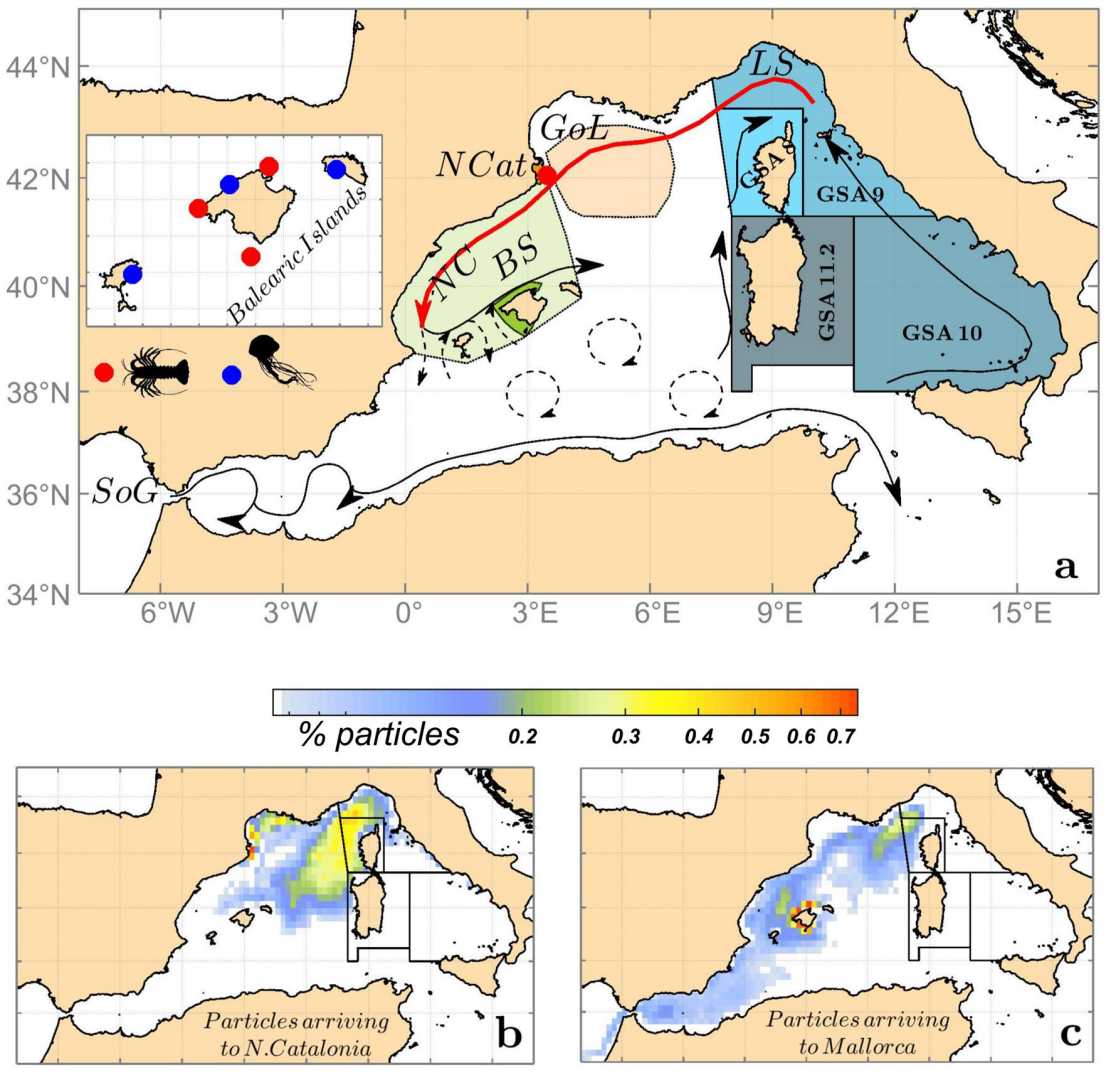
Average sea surface circulation in the western Mediterranean and biological time series locations. The lobster settlement index (LSI, red circles) belongs to different marine protected areas (MPAs) at two sites, one in the northernmost basin (NCat) and three in the Balearic Islands (North, NW and South Mallorca, green area and figure inset) (**a**). The jellyfish stinging index (JSI, blue circles) covered three tourist hot-spot locations in the Balearic Islands with different orientations and exposed to different water masses (E Ibiza, N Mallorca and S Menorca). Squares denote FAO Geographical Statistical SubAreas (GSAs) used to interpret the connectivity of LSI lobster series (see “Methods”). The Gulf of Lions (GoL, light orange area) and Balearic Sea (BS, light green area) indicate the areas where ocean-atmosphere fluxes were extracted for comparison against field records. A 10-year climatology of drifter sources (%) reaching either NCat (**b**) or Mallorca (**c**) is displayed as the particle location eight months before the observations (origin). Source geographic locations were aggregated in monthly density maps of $0.25 \times 0.25^\circ$. NC, Northern Current in red. LS, Ligurian Sea. SoG, Strait of Gibraltar. The figure and superimposed maps were created using “M Map: A mapping package for MATLAB”, v.1.4m (www.eoas.ubc.ca/~rich/map.html) in MATLAB v.R2010b (www.mathworks.com).

The Mediterranean Sea is a semienclosed basin and is considered a small-scale ocean. The western Mediterranean sub-basin is subject to well-identified circulation and production patterns, largely differing from the eastern sub-basin, mainly due to topographic constraints^35^. The excess evaporation in the basin is compensated with a continuous inflow of fresher Atlantic Waters (AW) through the Strait of Gibraltar (Fig. 1a). AW flows along the marginal southern and eastern continental slopes following an anticlockwise circuit, and AW is progressively transformed by atmospheric interaction into saltier and colder modified Atlantic water (MAW), particularly in the northernmost basin. From the Ligurian Sea toward the Balearic Sea, the Northern Current (NC) is the dominant geostrophic feature that closes the circulation cell at surface waters. The Gulf of Lions (GoL) is one of the few ocean regions where open-sea deep convection leads to deep or intermediate water formation, mainly occurring every winter. These phenomena are forced by ocean-atmosphere exchanges and persistent and cold N-NW winds^25^. The Balearic Sea is considered a particular water mass, sharing similar dynamics and features with other sub-basins, such as South China or the Caribbean Seas^36^. Through the Balearic Channels, there is a meridional exchange between new AW and colder, saltier, and productive MAW transported by the NC, forming an oceanographic density front, the Balearic front. Frontal emplacement is mainly driven by the blocking or exchange of water masses through Balearic channels. The meridional position of this front and channel exchanges are crucial to the marine ecosystem’s derived response around the islands^36,37^.

## Methods

### Simulation specifications and Lagrangian scheme

We used a state-of-the-art 3D regional climate simulation as the background hydrodynamic model, where a Lagrangian model is coupled to simulate drifting patterns and sources. Specifically, we used the Nucleus for European Modeling of the Ocean NEMO-MED36v75 (version 3.2) covering the whole Mediterranean basin for the period 2003-2013, using a horizontal resolution of $1/36^\circ$ ($\approx$ 2–3 km) and 75 z-vertical levels. For the simulations set carried out here, only the first 300 m were considered, and the domain was restricted to the western Mediterranean (Fig. 1). NEMO-MED36v75 successfully reproduces the main circulation features in the Mediterranean basin (surface circulation, coastal current shape and velocities, and deep convection) and mesoscale dynamics in the NW Mediterranean^38–40^.

**Figure 2:**
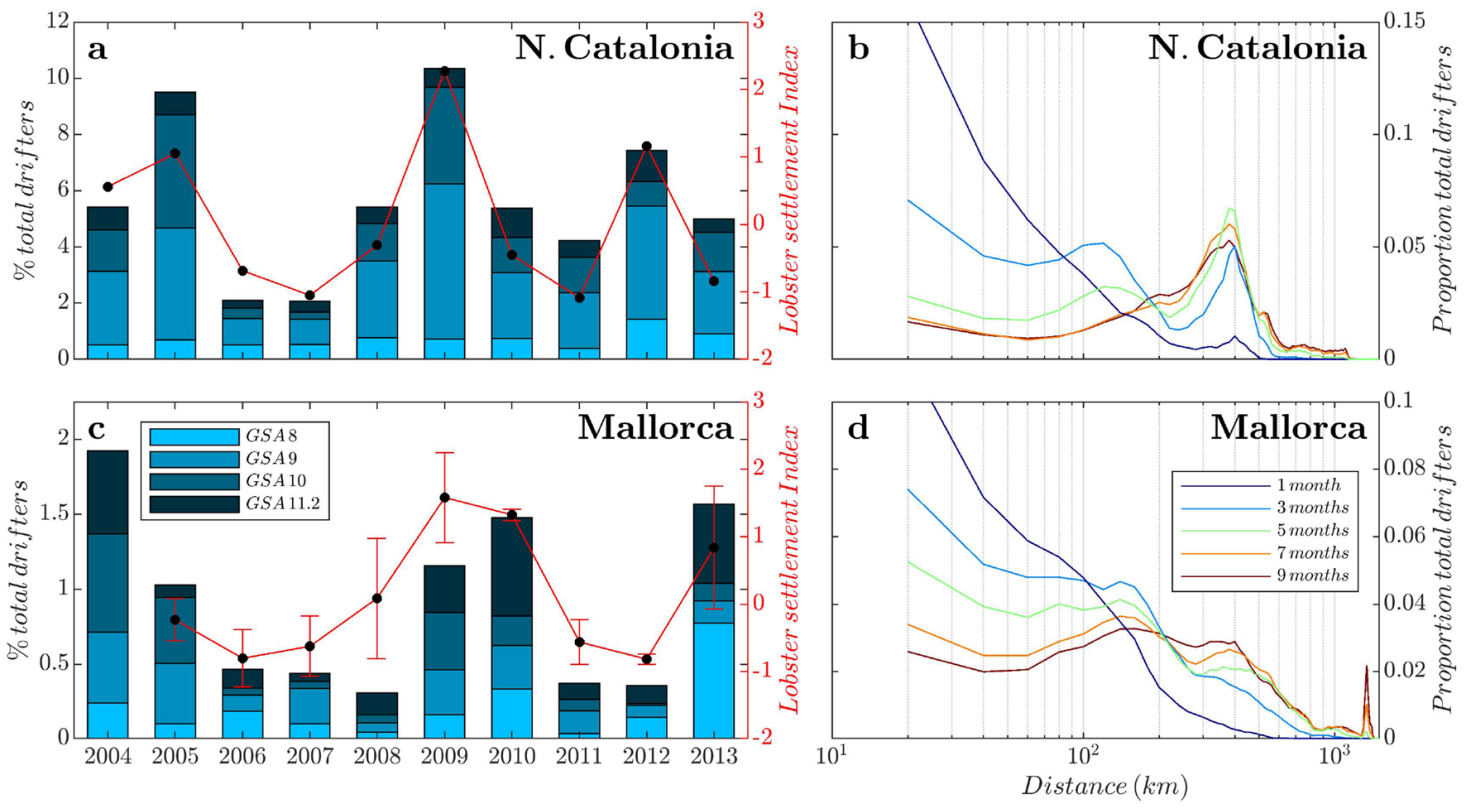
Spatiotemporal connectivity patterns and dispersal kernels. Comparison of observed LSI anomalies for NCat and Mallorca (red line, Panels **a** and **c**, errors are 1 SD) vs. percentage of individuals arriving from adult lobster habitat GSAs (0–200 m) (blue bars) (Fig. 1). The percentage of individuals was averaged over the settlement season (May to August) to compare with settlement field records. Correlations LSI vs. total % individuals from GSAs (8, 9, 10, 11.2): NCat ($r=0.89$; p-value $=0.0003$; $n=10$); Mallorca ($r=0.81$; p-value $=0.004$; $n=9$). Dispersal kernels were calculated for drifting times spanning from 1 to 9 months (panels **b**, **d**).

A spatially explicit individual-based model was used to simulate the dispersal and arrival of individuals along the western Mediterranean coasts. The model was forced with the 3D hydrodynamic model daily velocity fields using a customized version of the open source modeling tool ICHTHYOP^41^. As detailed biological information on the movement of these species through all their planktonic phase is lacking, passive Lagrangian particles (without buoyancy) were subjected to advective and diffusive processes that conditioned their horizontal and vertical movement in the water column.

To establish the appropriate number of virtual individuals to be released in a single simulation, multiple trials were conducted in which the number of released individuals were progressively increased (5000, 10,000, 15,000, 20,000, 50,000, 100,000, 125,000, and 150,000). The average and standard deviation of the results were calculated for each trial, and the point at which these statistics stabilized was determined^42^. Through this process, it was determined that a release of at least 100,000 individuals per simulation was appropriate ($p < 0.001$). Therefore, the simulations were set up with 134,000 individuals starting within the two closest cells to the shoreline ($\approx$ 5 km) with a randomly distributed vertical distribution throughout the Western Mediterranean basin. It was assumed that further repetition was not necessary to obtain unbiased results for each simulation. Backward-in-time trajectories of the drifters were simulated to assess the dispersal route and source areas. Each simulation covered 240 days (8 months) following the pelagic duration of the chosen species here^28,43^. The simulation set included 26 simulations per year, starting every year from the same odd weeks and covering 10 years, from 2004 to 2013 (260 simulations). Furthermore, the initial positions of the individuals were always the same in each simulation; thus, we ran 260 trajectories of the same individual.

Individuals were filtered from initial positions following available field records of LPDP, with ca. 1300 at North Catalonia (NCat) and 3000 (Mallorca) trajectories per location and simulation (Fig. 1a). Subsequently, simulation outputs were analyzed in two different ways:

1. Density maps of each site were computed summing all source positions of individuals using a grid of $1/4^\circ$ of horizontal resolution. Different simulations were gathered to reconstruct source monthly maps over the 10 years (120 maps) to increase the number of simulated individuals. Using a reference date at the 15th day of each month, we used moving 3-month windows (reference date ± 1.5 months) gathering 6 simulations for each density map, meeting a balance to include enough individuals/simulations but not reaching an excessive smoothing of oceanic processes. Different time windows were tested, obtaining similar results. Then, an empirical orthogonal function (EOF) analysis^44^ was applied to the set of density maps. We used the first EOF and principal components (PC) that explained the highest percentage of variability to extract the main spatiotemporal pattern of the source areas.
2. From each simulation, regarding connectivity for lobster settlement, we filtered the drifters at sources falling within lobster adult habitats (shallowest 200 m)^43^ and aggregated them using the geographic subareas (GSAs) of the FAO General Fisheries Commission for the Mediterranean. We computed and averaged the percentage of individuals from each GSA (Fig. 1a) during the lobster settlement season (May to August)^26^. In the absence of more complex biological data, we hypothesized that key atmosphere-ocean fluxes should explain a significant fraction of the simulation results, including their interannual variability. Subsequently, we tested these proposed driving mechanisms against the best available field records of LPDP.

**Figure 3:**
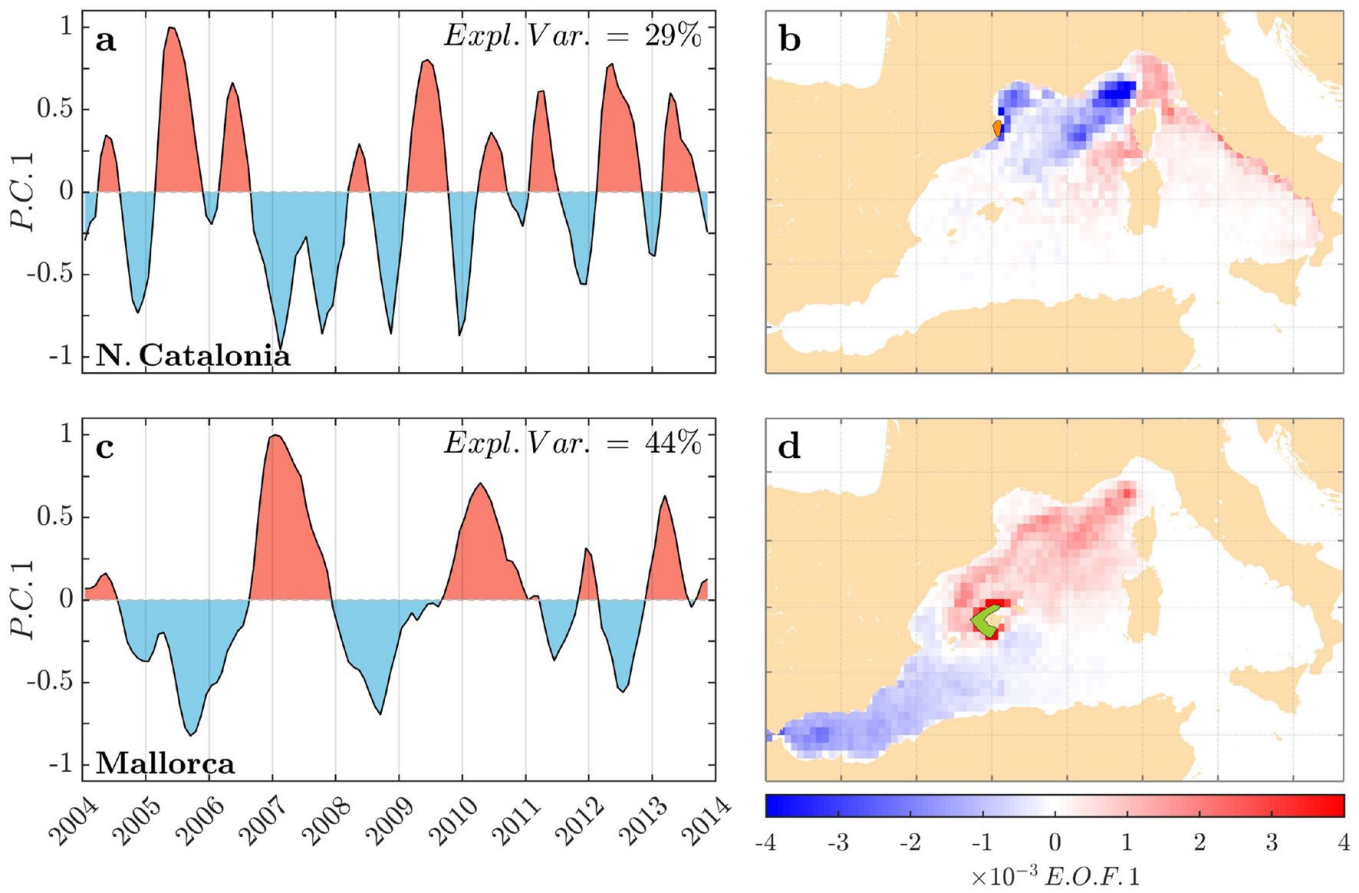
Prevailing sources and interannual patterns of LPDP arriving at the target areas after eight months. Temporal (**a**,**c**) and spatial (**b**,**d**) representation of the first principal component (PC1) of empirical orthogonal function (EOF) analysis conducted on source areas of individuals based on 120 monthly origin maps (2003–2014) (more details in “Methods”). The results are for N. catalonia (**a**,**b**) and Mallorca (**c**,**d**). The total explained variance was 29% for N. Catalonia and 44% for Mallorca. Positive PC and EOF values are in red, and negative values are in blue. Original source maps can be reconstructed using the climatology (Fig. 1b,c) and adding the product of PC1 by EOF values. Therefore, intensified influence as source areas emerges when blue/red areas (from EOF value) and PC value match (blue/red). The figure and superimposed maps were created using “M Map: A mapping package for MATLAB”, v.1.4m (www.eoas.ubc.ca/~rich/map.html) in MATLAB v.R2010b (www.mathworks.com).

**Figure 4:**
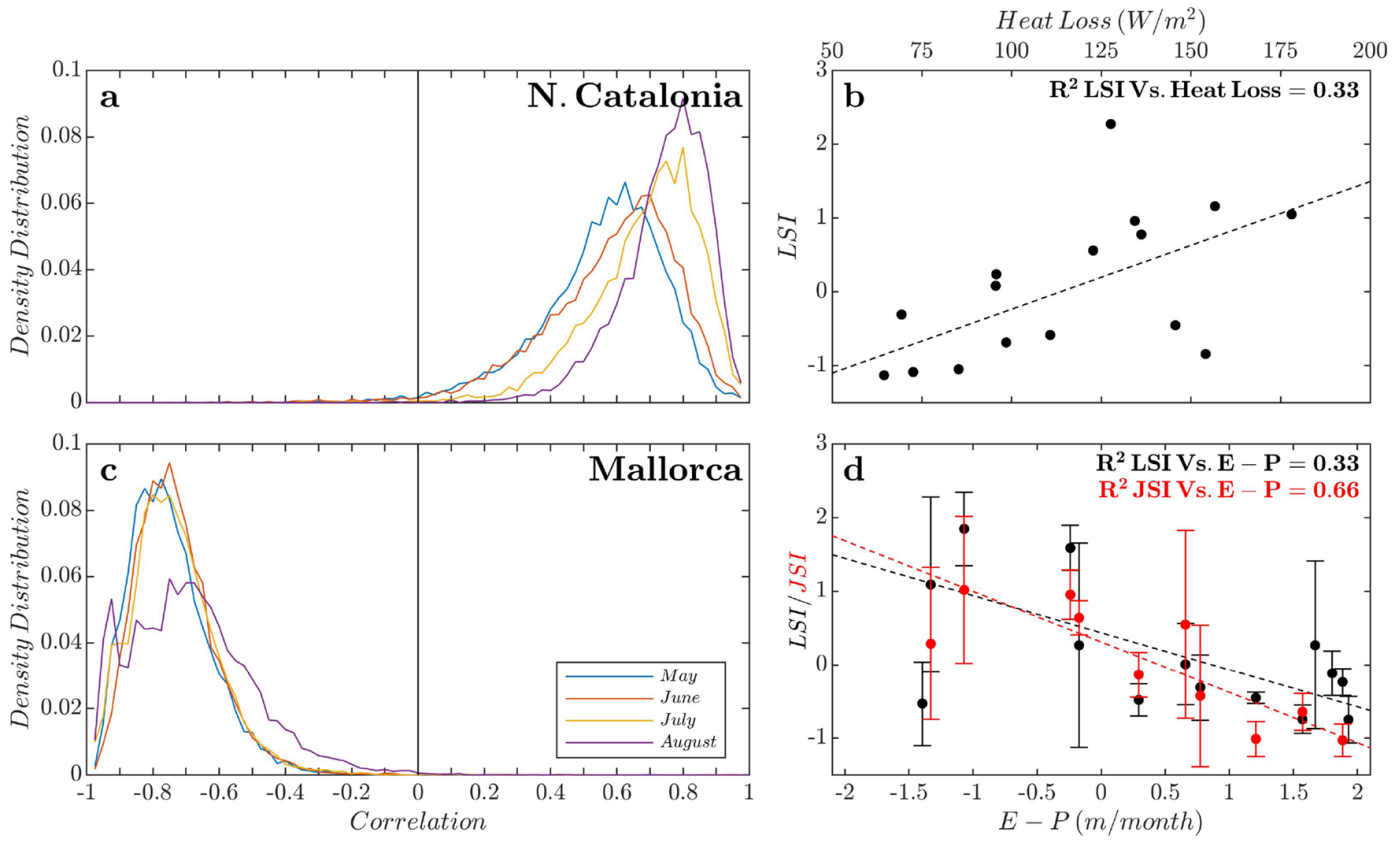
Atmosphere-ocean coupling driving dispersal patterns and proposed mechanisms explaining LPDP interannual variability. Density distribution of the Pearson correlation coefficients between ocean-atmosphere fluxes and PC1 (Fig. 3a,c) for summers/settlement season of jellyfish and lobster, which was obtained by bootstrap resampling (10,000 times) for the two sites: (**a**) Heat loss (HL) vs. PC1 for North Catalonia (NCat) (Fig. 3a). (**c**) (Evaporation-precipitation, E−P) vs. PC1 for Mallorca (Fig. 3c). Linear relationship of the atmosphere-ocean fluxes and LPDP field records for both sites, Lobster Settlement Index (LSI) and Jellyfish Stinging Index (JSI). (**b**) For NCat, HL vs. LSI ($R^{2}=0.33$; p-value $=0.021$, $n=16$). (**d**) In Mallorca, (E−P) vs. LSI ($R^{2}=0.33$; p-value $=0.003$, $n=14$) and (E-P) vs. JSI ($R^{2}=0.66$; p-value $=0.005$, $n=10$). More details in Supplementary Table S2, Supplementary Information). We found synchronic patterns for both LPDP: LSI vs. JSI ($R^{2}=0.65$; p-value $=0.005$, $n=10$).

The simulation was originally forced by ARPERA atmospheric fields^45^ but in this case, we used the fluxes from the longer ERA5 atmospheric reanalysis to extend the analysis out of the simulation period, firstly checking the similar behaviour of both atmospheric reanalysis^46^, considering positive fluxes from the ocean toward the atmosphere. We extracted the monthly averaged fluxes (2001–2018) of (i) the ocean heat loss, using here the average during winter months (JFM) from the GoL area^25^ (Fig. 1a, light orange) and (ii) evaporation-precipitation budget in spring (April) from the Balearic Sea (Fig. 1a, light green).

The strength of the link between the atmosphere-ocean fluxes and main dispersal patterns via the first PC (PC1) was also assessed through robust correlation analysis. We quantified the probability density distribution of Pearson correlation coefficients obtained by bootstrap resampling. This analysis involved random pairwise sampling with replacement, where each time series was resampled 10,000 times. The number of elements in each bootstrap sample equals the number of elements in the original dataset. Detailed results of this analysis can be found in the SI (Supplementary Table S2).

### Lobster Settlement Index

The Lobster Settlement Index (LSI) was assessed at two main sites, including one MPA at NCat and three MPAs at Balearic Island (north, west and south of Mallorca) (Fig. 1a). Early benthic juveniles were recorded once a year during summer from 2001 to 2018, and more specific details can be found in reference^27^.

No live animals were used in this study; accordingly, ethical approval was not required.

### Jellyfish stinging index

We identified summers with a high abundance of jellyfish arriving at the Balearic shores. Here, we proposed an index based on the number of first aids given to bathers due to jellyfish stings as a proxy of arriving events of *P. noctiluca*, the main stinging species in this basin^21,47^. To this aim, we used a database originally developed by the Regional Government Emergency Area of the Balearic Islands. Lifeguard daily reports of first aid and causes in beach locations were compiled in the database. We identified and extracted three coherent time series without gaps corresponding to three beach sites with high potential jellyfish impact on the bathers, one on each major island of the Balearic archipelago: Ibiza, Mallorca and Menorca (Fig.1a). Each time series comprised the number of first aids, including jellyfish stings aggregated over the tourism peak season (July August), covering the 2009–2018 period. To avoid biases, the number of stings was normalized over the number of first aids excluding the stings as a proxy of the number of bathers at each beach. Furthermore, we computed each site stinging series annual anomaly to avoid bias due to local factors.

### Consent to participate

The authors voluntarily agree to participate in this research study.

## Results and discussion

### Sources and dispersal routes in the NW Mediterranean

We show that NCat and Mallorca shores shared source areas in the northernmost basin, following the NC path from the Ligurian Sea (Fig. 1b,c). However, Mallorca shore sources also extended along the entire southwestern basin from the Strait of Gibraltar (Fig. 1c). The northeastern GSAs (GSAs 8, 9, 10 and 11.2) showed a large match and tight correlations with the observed LSI at both sites, although this area contributed to the replenishment of NCat and Mallorca only at low percentages, with peaks ca. 10% and 1.5%, respectively. Other GSAs showed a higher potential contribution as source areas (Fig. 1b,c; Supplementary Fig. S1), although they showed low or even negative correlations with LSI (Supplementary Fig. S1).

Our simulations unveiled that the Ligurian Sea acts as a source (origin) of drifters reaching NW Mediterranean shores via NC, also shaping drifter routes whose pelagic lifespan is shorter than 8 months (Fig. 2b,d). Therefore, the Ligurian Sea may act as a “pelagic nursery area” *sensu* Kough et al.^48^ due to physical processes known to trap plankters long enough to foster productivity for high trophic levels, including marine mammals, seabirds and fish^49^. This partly explains why this area has been identified as a biodiversity hot spot in the Mediterranean and declared the Pelagos Sanctuary for Mediterranean marine mammals^50^.

### Ocean–atmosphere coupling mold dispersal and connectivity

Source areas feeding NCat and Mallorca shores are closely linked with the ocean–atmosphere system playing out in the North Atlantic, the influence of which shapes interannual hydrographic variations^24^ and dispersal patterns in the western Mediterranean. For NCat, a marked seasonal modulation in the source area was captured. During spring and summer (from May to August), variability in source areas was mainly ascribed to the Ligurian Sea and to both current branches encircling Corsica (GSA8, Fig. 1a) feeding the NC (Fig. 3a,c in red). At interannual scales, principal component 1 (PC1, temporal pattern) for spring and summer mainly portrayed the ocean heat loss winter variability in GoL (Fig. 4a). Harsh and windy winters yield intense ocean heat loss around the GoL, promoting deep convection and intermediate or deep-water formation. These processes enhance the strength of the NC^25,51^, ultimately increasing the contribution of the northeastern shores as source areas (Figs. 2a,b and 3; Supplementary Fig. S2). Surprisingly, we found that these conditions, in particular the intense heat loss, were concurrent with major lobster settlement peaks in NCat, as shown by the close correlation between winter heat loss and LSI ($R ^{2}=0.33$; p-value $=0.020$; $n=16$), whereas lower settlement events occurred after warm winters, when heat loss declined (Fig. 4b).

Our simulations uncovered dipole sources and dispersal routes feeding Mallorca shores, involving (i) the circulation cell of MAW encompassing NC, the Balearic Sea and the Ligurian Sea (red) and (ii) the inflowing fresher southwestern AW (blue) (Fig. 3d). A marked interannual signal (Fig. 3a) suggests a seesaw-like effect promoting an alternance in main source areas, ca. 2–3 years. This pattern mirrors the water balance (evaporation–precipitation, E−P) in spring in the Balearic Sea (Fig. 4c). An excess of evaporation (E > P) in this sub-basin drives the northward progress of fresher AW surpassing the Balearic channels. Thus, AW acts as the main source of arriving drifters in the Balearic Islands (negative PC1 and spatial values, blue in Fig. 3c,d) while blocking the arrival of MAW and constraining the contribution of northern sources (Supplementary Fig. S2). In contrast, rainy springs (P > E) increased the influence of productive MAW and northern source areas (positive PC1 and spatial values, red in Fig. 3c,d; Supplementary Fig. S2), ultimately resulting in marked peaks of both LPDS arrivals, as suggested by LSI and JSI field records (Fig. 4d). We showed evidence of a synchronic interannual pattern involving different LPDS (LSI vs. JSI, $R ^{2}=0.65$; p-value $=0.005$, $n=10$). This synchrony in coastal arrival, explained largely through physical forces, supports the observed association between stinging jellyfish and lobster phyllosoma, which may use the umbrella of the jellyfish as a shelter, food source and transport vector^52^.

### A mechanistic explanation of the interannual variability in western Mediterranean metapopulations

Our approach allowed us to mechanistically test our hypotheses, first unveiling the pervasive role of Ligurian Sea-NC and AW as prevailing dispersal routes of the LPDP in the W Mediterranean Sea (Figs. 1 and 3). Second, the dispersal patterns of different species shared with the LPDP in the W Mediterranean were further modulated at the interannual scale by atmospheric–ocean coupling, but the latter responded to a site-specific atmospheric driver (Fig. 4). This approach opens opportunities for understanding the processes and scales shaping metapopulation structure and dynamics and hints to test similar hypotheses in other marginal seas and semienclosed basins to boost conservation-management policies for marine resources.

The bloom frequency of *P. noctiluca* in the western Mediterranean apparently has increased along with environmental changes in the region experienced in recent decades^33^. However, little synoptic information exists on its population structure and dynamics in this basin^21,31^, although this information is essential for jellyfish management actions, particularly where economic activities are threatened. Current knowledge suggests that a single mauve stinger jellyfish population exists in the whole basin^53^ and that interannual variability in stranding is partly coupled with climate^32,33^ and stochastic variations^30,54^. Short-term forecasting can effectively simulate local jellyfish stranding events^54,55^; however, we show that a comprehensive description and potential forecast of jellyfish blooms at larger scales cannot be achieved without fully resolving source areas and dispersal routes during the whole lifespan of the species^56^. The explanatory mechanisms proposed here open the possibility for potential forecasting of swarm dispersal and stranding events, stressing the potential use of North Atlantic climate variability for the W Mediterranean (Fig. 4). Furthermore, these findings enable a future design of research efforts, e.g., description of processes at retention areas, to disentangle the relative role of physical and biological components on the interannual variability of these blooms.

Our findings on spiny lobster settlement offer a mechanistic explanation of its interannual variability and emphasize that fisheries management areas and political borders do not fit the metapopulation dynamics of species with the LPDP, particularly in marginal seas with several riverine countries^48,57^. Hence, these results are essential for tailoring the managing actions of spiny lobster fisheries in the Western Mediterranean. Genetic studies suggest a common lobster larval pool for the northernmost basin in the NW Mediterranean, which is supported by our simulations that further unveiled a potential influx of individuals from southern or Atlantic sources feeding the Balearic Islands population^58,59^. Our results bear vast implications for conservation and resource management policies, as the connectivity patterns uncovered by our simulations showed that on the NW Spanish coast, MPAs might benefit from spiny lobster population recovery in the Sardinia area (GSA 11.2 in Figs. 1a and 2a, c; Supplementary Figs. S1, S2)^60^, thus stressing the need for wide-scale multinational efforts to develop effective resource management and conservation policies. Ignoring source areas will jeopardize the sustainable management of the already threatened spiny lobster population of the Mediterranean Sea.

Our results on the physical influence on LPDP dispersal patterns set a baseline to connect physical forcing with future climate trends. We demonstrated that winter conditions in the GoL area are crucial for LPDP dispersal and connectivity, thus explaining interannual variations. Projections for 2030^61^ suggest oceanographic shifts leading to an increase in (i) the strength and duration of deep convection and (ii) an enhancement of mesoscale activity and NC circulation due to meridional wind component intensification. These changes may affect the metapopulation features of species with LPDP in the northwestern Mediterranean and therefore should be considered in management scenarios.

## Supplementary Information

Supplementary Information.

## Data Availability

The data that support the findings of this study are available from the corresponding author upon reasonable request.
