# Supplementary material for: Atmospheric–ocean coupling drives prevailing and synchronic dispersal patterns of marine species with long pelagic durations
Source: Sci Rep. 2023 Feb 9;13:2366. doi: 10.1038/s41598-023-29543-7 (PMC9911598; doi:10.1038/s41598-023-29543-7)
Supplement: Supplementary file 1 — Supplementary Information. [file 41598_2023_29543_MOESM1_ESM.pdf]

Supplementary Information for

Atmospheric-ocean coupling drives prevailing and synchronic dispersal patterns of marine species with long pelagic durations

Eduardo Ramírez-Romero, Ángel Amores, David Díaz, Anabel Muñoz, Ignacio A. Catalán, Juan C. Molinero and Andrés Ospina-Álvarez

Corresponding author: Andrés Ospina-Álvarez
E-mail: andres.ospina@csic.es

This PDF file includes:

Figs. S1 to S2
Tables S1 to S2

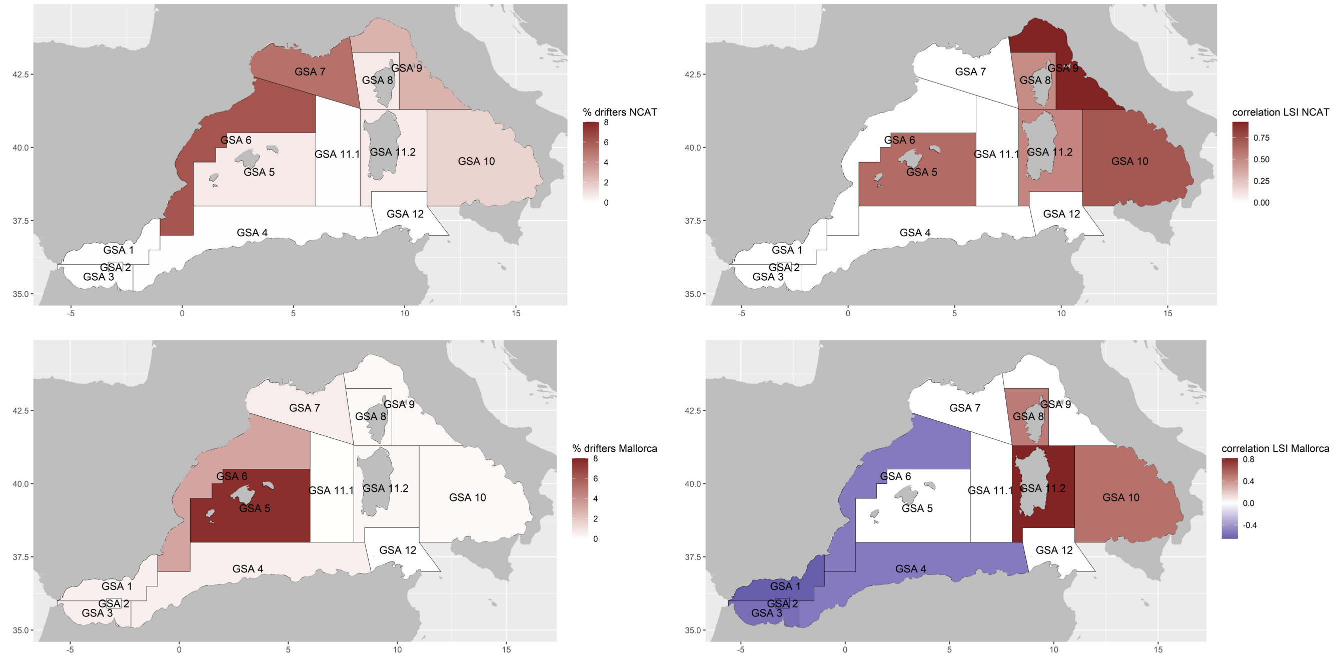

Fig. S1. Percentage of drifters arriving from adult lobster habitat (0-200 m) from each Geographical Sub Areas (GSAs, Fig. 1). The percentage of drifters was averaged over the settlement season (May to August) to compare with settlement field records: a- for NCat and c- for Mallorca. Significant Pearson correlations ($p < 0.1$, $n = 10$) are shown, Lobster Settlement Index LSI vs. % drifters from each GSAs, for b-NCat and d-Mallorca. The figure was created with R v.4.2.0 (https://cran.r-project.org) packages: "ggplot2" v.3.2.113 (https://ggplot2.tidyverse.org) and "ggmap" v.3.0.029 (https://github.com/dkahle/ggmap).

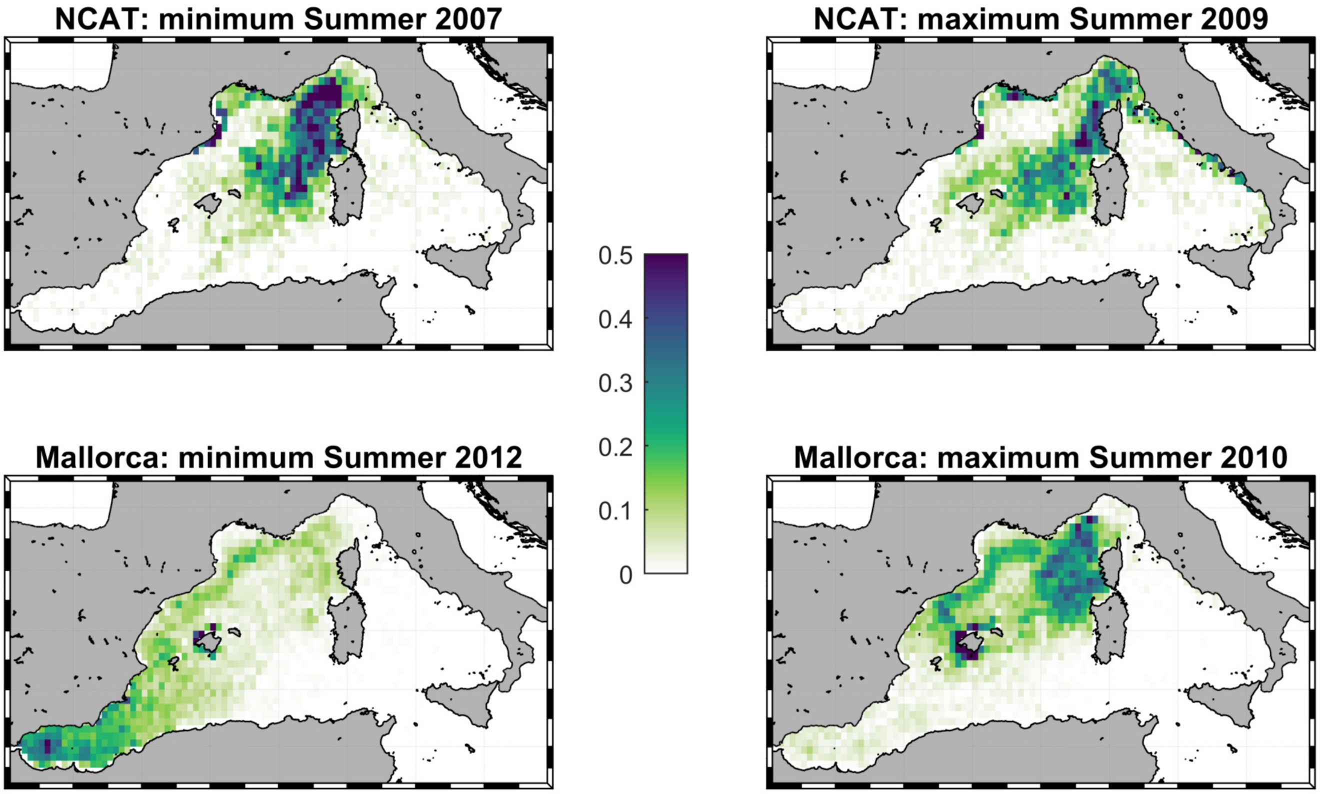

Fig. S2. Drifter sources (%) reaching either NCat (a,b) or Mallorca (c,d) during different maxima and minima the particle location eight months before the observations (origin). Source geographic locations were aggregated in monthly density maps of $0.25x0.25°$. The figure was created with R v.4.2.0 (https://cran.r-project.org) packages: "ggplot2" v.3.2.113 (https://ggplot2.tidyverse.org) and "ggmap" v.3.0.029 (https://github.com/dkahle/ggmap).

Table S1. Percentage of drifters arriving from adult lobster habitat (0-200 m) and significant Pearson correlations depicted in Figure S1 for both sites (n=10)

GSA	% particles NCat	correlation vs LSI NCat	p-value	% particles Mallorca	correlation vs LSI Mallorca	p-value
GSA 1	0,02			0,54	-0,65	0,03
GSA 3	0,01			0,60	-0,57	0,05
GSA 4	0,04			0,54	-0,53	0,07
GSA 5	0,73	0,62	0,03	7,64		
GSA 6	6,24			3,21	-0,53	0,07
GSA 8	0,72			0,21	0,48	0,09
GSA 9	2,72	0,93	0,00	0,23		
GSA 10	1,57	0,71	0,01	0,20	0,53	0,07
GSA 11.2	0,68	0,51	0,07	0,27		

Table S2. Details on density distribution of the Pearson correlation coefficients between ocean-atmosphere fluxes and PC1 (Fig. 4 a,c) for summers/settlement season of jellyfish and lobster, which was obtained by bootstrap resampling (10,000 times) for the two sites.

Site	Correlation	mean correlation	p-value	standard deviation
NCAT	HL vs PC1-May	-0,58	0,041	0,02
NCAT	HL vs PC1-June	-0,62	0,029	0,02
NCAT	HL vs PC1-July	-0,71	0,010	0,02
NCAT	HL vs PC1-August	-0,77	0,005	0,02
Mallorca	(E-P) vs PC-May	-0,75	0,006	0,13
Mallorca	(E-P) vs PC-June	-0,74	0,008	0,13
Mallorca	(E-P) vs PC-July	-0,75	0,007	0,13
Mallorca	(E-P) vs PC-August	-0,69	0,012	0,18
